# Communication on analgesia and sedation: parents’ opinions in six pediatric ICU in northern Italy: Eleonora Monaco

**DOI:** 10.1093/eurpub/ckac131.184

**Published:** 2022-10-25

**Authors:** M Martinato, PC Fazio, R Sagredini, G Pagano, S Ferrario, A Conio, E Monaco, D Gregori, A Amigoni, MC Mondardini

**Affiliations:** 1 Department of Cardiac Thoracic Vascular Sciences and Public Health, Università degli Studi di Padova, Padua, Italy; 2 Department of Statistics, Informatics, Applications, Università di Firenze, Firenze, Italy; 3 PICU, University Hospital Padua, Padua, Italy; 4 PICU, IRCCS Materno Infantile Burlo Garofolo, Trieste, Italy; 5 PICU, University Hospital Verona, Verona, Italy; 6 PICU, ASST Fatebenefratelli Sacco, Ospedale dei Bambini Vittore Buzzi, Milan, Italy; 7 PICU, Azienda Ospedaliero-Universitaria Città della Salute e della Scienza, Turin, Italy; 8 PICU, Azienda Ospedaliero-Universitaria di Bologna IRCCS Policlinico, Bologna, Italy

## Abstract

**Introduction:**

Communication between healthcare professionals and parents regarding analgesic and sedative treatments for seriously ill children is challenging. Although appropriate information may help parents during admission to the pediatric intensive care unit (PICU), some areas of communication may be missed.

**Objectives:**

To explore and describe the opinions of parents of children admitted to PICU about analgosedation, information received about it and its potential adverse effects, and suggestions for improving the comfort of hospitalized children.

**Methods:**

Parents’ opinions were collected in six PICUs in northern Italy. Parents of children who were hospitalized for more than 48 hours and required analgesia and sedation were asked to provide opinions on the quality of information with respect to the treatments used, possible short- and long-term sequelae, satisfaction with efficacy, environmental factors perceived as disturbing, and suggestions for improving children’s comfort.

**Results:**

Forty-eight parents participated. Information about analgosedation was rated, as was its effectiveness. Parents pointed out some interesting suggestions to improve their children’s comfort during their hospitalization in PICU. On the other hand, it was found that information about possible complications, withdrawal syndrome, delirium, or difficulties in obtaining the desired level of sedation was often provided poorly or inadequately. In addition, wide differences were found between centres, particularly with regard to analgosedation side effects and withdrawal syndrome.

**Conclusions:**

Parents should receive early explanations of these potential complications. Even considering the limited number of participating PICUs, distributed exclusively in northern Italy, and the small number of participants, this is the first data collection focused on communication between health professionals and parents of children undergoing analgosedation treatment in pediatric intensive care units.

**Key messages:**

• This is the first data collection focused on communication between health professionals and parents of children undergoing analgosedation treatment in pediatric intensive care units.

• Information about possible complications, withdrawal syndrome, delirium, or difficulties in obtaining the desired level of sedation was often provided poorly or inadequately.

